# Hepatic zinc deficiency dampens the acute phase response in patients with alcohol-associated hepatitis

**DOI:** 10.3389/fimmu.2025.1642163

**Published:** 2026-02-26

**Authors:** Scott A. Read, Mehdi Ramezani-Moghadam, Brian S. Gloss, Romario Nguyen, Benjamin Woodham, Vincent Lam, Lawrence Yuen, Jimin Yoon, Liang Qiao, Thomas Tu, Jacob George, Matthew D. Shoulders, Golo Ahlenstiel

**Affiliations:** 1 Blacktown Clinical School, Western Sydney University, Blacktown, NSW, Australia; 2 Blacktown Hospital, Western Sydney Local Health District (WSLHD), Blacktown, NSW, Australia; 3 Storr Liver Centre, The Westmead Institute for Medical Research, The University of Sydney and Westmead Hospital, Westmead, NSW, Australia; 4 Westmead Research Hub, Westmead Institute for Medical Research, Westmead, NSW, Australia; 5 Westmead Hospital, Western Sydney Local Health District, Westmead, NSW, Australia; 6 Department of Chemistry, Massachusetts Institute of Technology, Cambridge, MA, United States; 7 Koch Institute for Integrative Cancer Research at Massachusetts Institute of Technology (MIT), Cambridge, MA, United States; 8 Broad Institute of MIT and Harvard, Cambridge, MA, United States

**Keywords:** acute phase response, alcohol-associated hepatitis, alcohol-related liver disease, metallothionein, zinc, zinc deficiency, zinc signature

## Abstract

**Introduction:**

Zinc deficiency affects ~17% of the population globally, contributing to deficits in growth, metabolism and immunity. Serum zinc is greatly reduced in alcohol-associated hepatitis, driven by hepatic dysfunction and poor zinc retention. While zinc is an essential micronutrient with many beneficial anti-inflammatory and anti-oxidant properties, its role in the progression of alcohol-related liver disease (ALD) remains uncertain.

**Methods:**

To identify broad transcriptomic responses to zinc, 11 publicly available datasets were examined to generate a transcriptomic zinc signature. Zinc signature genes were validated in vitro using primary immune cell and hepatocyte models supplemented with zinc or depleted of zinc using a S100A12 conjugated resin. The role of zinc deficiency in alcohol-associated hepatitis was examined bioinformatically, using large publicly available datasets, and confirmed *in vitro*.

**Results:**

A nine gene zinc signature consisting primarily of metallothonein genes identified hepatic zinc deficiency among ALD patients that was associated with a down-regulation of the acute phase response pathway (e.g., *SAA1, CRP* and *C9* genes). *In vitro* studies using hepatocyte and immune cell cultures depleted of zinc demonstrated that macrophage induction of the acute phase response by LPS was dampened by zinc depletion.

**Discussion:**

Together, these data suggest that hepatic zinc stores among patients with alcohol-associated hepatitis are reduced, resulting in deficient acute-phase responses. Increasing hepatic zinc stores via supplementation and dietary modulation may improve acute responses to infection, and thus, long term outcomes in this patient group.

## Graphical Abstract

**Figure:**
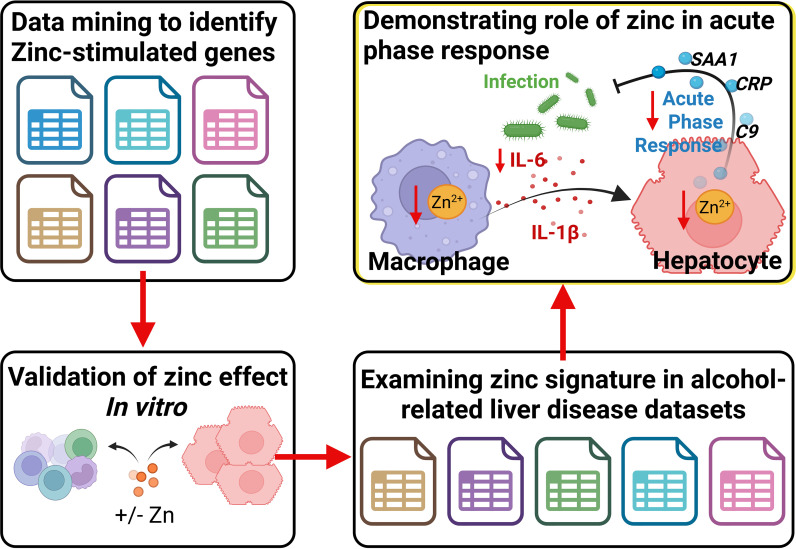

## Introduction

Zinc is an essential trace element, and a structural constituent of ~10% of the human proteome acting as a catalytic component in over 300 enzymes and a structural component in ~750 transcription factors (1, 2). Accordingly, zinc plays a significant role in growth, sexual maturation, metabolism and immunity (3). Almost 1 in 5 individuals globally are at risk of zinc deficiency, a condition that disproportionately affect vulnerable populations affected by food insecurity and/or chronic illness (4). Impaired immune system development and homeostasis is one of the most apparent symptoms of severe zinc deficiency, with stricken individuals demonstrating significant thymic atrophy and lymphopenia (5). Consequently, zinc deficiency is associated with recurrent infections (6), resulting from major defects in immune cell maturation (7) and functionality (8).

Zinc deficiency is particularly common among individuals with chronic liver disease (CLD) (9), resulting from poor zinc absorption (10) and impaired retention due to low albumin production in the liver (11), the major carrier of zinc in blood. Accordingly, normalization of serum zinc has been shown to significantly improve markers of liver function (*e.g.*, bilirubin, alkaline phosphatase and albumin) in patients with cirrhosis, while improving survival and reducing incidence of hepatocellular carcinoma (HCC) (12, 13). Alcoholism can exacerbate zinc deficiency independent of liver disease by promoting increasing urinary zinc excretion (14) and decreasing intestinal absorption (15). Alcohol-associated hepatitis (AH) is a severe condition caused by excessive alcohol intake, characterized by rapid onset of jaundice, and elevated liver stress markers including bilirubin and aspartate aminotransferase (AST). Due to excessive alcohol intake over the course of months to years, combined with liver dysfunction that is common among these patients, AH represents one of the most zinc deficient liver diseases in both blood and liver (16, 17).

Due to ease of sampling and clinical diagnosis, zinc is almost exclusively quantified from serum where it represents only 0.1% of total systemic zinc. Serum zinc can also poorly reflect tissue zinc concentration, that can be disproportionately affected by zinc deficiency (18). To understand the role that zinc deficiency plays in the pathology of liver disease, accurate assessment of zinc concentrations within diseased tissue is essential. To address this problem, we hypothesized that zinc status within tissue can be estimated based on zinc-stimulated gene (ZSG) expression. Using publicly available datasets, we generated and validated a gene signature score to estimate zinc content within liver tissue from patients with chronic liver disease, focusing on AH. The transcriptomic zinc signature score was used to query large datasets of AH liver tissue transcriptomes to better understand how zinc deficiency in the liver contributes to the pathogenesis of AH. Bioinformatic analysis suggested that zinc is a major driver of the acute phase response in the liver. As macrophages and hepatocytes are the primary drivers of the acute phase response, we validated the role of zinc *in vitro* using primary hepatocyte and macrophage cultures. These data suggest that zinc deficiency in AH specifically dampens the macrophage acute phase response to infection and has potential to significantly worsen long-term outcomes by making patients vulnerable to life-threatening infections.

## Materials and methods

### Gene expression dataset collection and analysis

The National Center for Biotechnology Information (NCBI) Gene Expression Omnibus (GEO) and PubMed were queried using the terms zinc, TPEN (N,N,N’N’-tetrakis (–)[2-pyridylmethyl]-ethylenediamine), treatment, depletion, supplementation, and deficiency. A total of 10 zinc treatment and three zinc depletion datasets were identified, of which nine and two were analyzed, respectively, based on dataset quality and availability. Datasets (19–28) were analyzed using the R statistical environment (29) as outlined in **Supplementary Figure S1**. Briefly, array expression data was logged and quantile normalized as required followed by dataset-specific filtration based on log expression data. Gene count data from RNA sequencing platforms underwent dataset-specific filtration, followed by normalization and dispersion estimation using EdgeR. Differential gene expression was evaluated using the Empirical Bayes Statistics for Differential Expression (eBayes) for array data and the Generalized Linear Model Quasi-Likelihood F-Test (glmQLF) for sequencing data using Benjamini-Hochberg method multiple testing correction to control for false discovery rate (FDR) (30) as we have done before (31). Differential gene expression performed by Palmer et al. (32) and Tuomela et al. (33) were additionally used in our analysis. Cell types were examined separately in all datasets containing more than one cell type (GSE39316, GSE39330) except for dataset GSE25167 where HaCat and Sk Mel-28 cell transcriptomes (n=1 each) were combined to allow statistical comparison between mock and zinc treated samples. ZSGs were defined by a significant (p<0.05) ≥1.5-fold induction in gene transcription following zinc treatment or conversely, a ≤1.5-fold reduction following zinc depletion.

AH transcriptomic datasets GSE28619 (34), GSE143318 (35), GSE155907 (36), GSE142530 (37) and GSE94417 (38) were analyzed as above. Datasets GSE94397, GSE94399 and GSE103580 were merged and batch corrected using ComBat (https://doi.org/10.18129/B9.bioc.sva) on quantile normalized data (39). Data access to the DbGaP study phs003112.v1.p1 (40) was approved by the National Institute of Health (Project #31612). For DbGaP study, fastq files and sample metadata were downloaded using SRAtools (v3.0.0), Library sequencing quality was determined using FastQC (Babraham Bioinformatics). Illumina adaptor sequence and low quality read trimming (read pair removed if < 20 base pairs) was performed using Trim Galore! (Babraham Bioinformatics: www.bioinformatics.babraham.ac.uk/). STAR 52 was used to align reads to human genome hg38 using ENSEMBL gene annotations as a guide. Read counts data corresponding to ENSEMBL gene annotations were generated using HTSeq (41). Bioinformatics processing and analysis pipelines have been added to https://github.com/Scotch25/ZincALDManuscript.

### Selection of zinc signature genes

Zinc-stimulated gene expression was collated from 11 datasets (19–26, 28, 32, 33), with ZSGs defined as being up-regulated ≥1.5 fold, p<0.05 (**Supplementary Table S1**). Genes that were up-regulated by zinc in ≥5 of 11 datasets were included in the preliminary zinc signature. A cutoff of 5 datasets, as opposed to 6 (representing >50% of datasets examined) was chosen to account for a lack of ZSG expression data within some datasets (**Supplementary Table S1**).

### Sample collection

Blood samples were collected from healthy volunteers at Westmead Hospital and the Westmead Institute for Medical Research. Liver tissue was obtained during hepatocellular carcinoma resection or bariatric surgery at Westmead and Blacktown Hospitals, respectively. All work was performed in accordance with both the Declarations of Helsinki and Istanbul. Ethics approval was obtained from the Sydney West Area Health Service and University of Sydney (HREC2002/12/4.9 (1564) and HREC/17/WMEAD/552 (5450)). Informed consent was obtained for all subjects.

### Primary and continuous cell isolation and culture

Peripheral blood mononuclear cells (PBMCs) were isolated using Ficoll Paque Plus (GE Healthcare) density gradient separation. PBMCs were cultured in RPMI medium containing 10% fetal calf serum at 37 °C with 5% CO_2_. To generate macrophages, 5 x 10^5^ monocytes were isolated from PBMCs using CD14 microbeads and the AutoMacs Pre Separator (Miltenyi Biotec). Cells were differentiated in RPMI medium plus 10% fetal calf serum and 50 ng/mL M-CSF over 7 days, changing the media on day 3 (42).

Liver organoid-based cell monolayers were generated from human liver tissue collected from liver biopsies/resections. Tissue was minced and digested with collagenase IV (1mg/ml) for 30 minutes at 37 °C after which cells were pushed through a 100 µm cell strainer and centrifuged at 50 x g for 5 minutes. The cell pellet representing concentrated hepatocytes was harvested, cells were resuspended in expansion media and the 50 x g centrifugation step was repeated twice. Hepatocytes were resuspended in Matrigel and organoid expansion media was added (media formulations available in **Supplementary Tables S2, S3**). Following sufficient expansion of liver organoids (3–5 days post-splitting while organoids are actively expanding but still relatively small), organoids were removed from matrigel using cold PBS, then digested with TrypLE for 3 minutes at 37 °C. The single cell suspension was added to a collagen coated 48 well plate in expansion media until cells reached 80-90% confluency (**Supplementary Figure S2**). Expansion media was replaced with differentiation media for 3 days prior to treatment with zinc supplementation/depletion media. Organoid cultured were grown at 37 °C with 5% CO_2._

Huh-7 cells were cultured in DMEM medium containing 10% fetal calf serum at 37 °C with 5% CO_2_. All experimental treatments were performed at ~80% confluence.

### In vitro zinc treatment and depletion

Zinc depletion of media was performed as previously described (27). Briefly, media was incubated at room temperature, rocking overnight with S100A12 zinc-binding resin at a ratio of 80 µl resin: 1 mL culture media. Resin was pelleted by centrifugation at 400 x g for 5 minutes and media was removed for immediate use. Following removal of culture media and a single wash with PBS, zinc-depleted (ZnDep) media was added to cells. For all zinc treatment and repletion experiments, 50 µM ZnSO_4_ was added to media.

### Quantitative PCR

RNA was extracted using the Favorgen Total RNA kit and cDNA was synthesized using MMLV reverse transcriptase (Promega). Gene expression was measured using VIC and FAM conjugated Taqman probes or gene specific primers and TaqMan Fast Advanced MasterMix (Thermo Fisher Scientific). A full list of primers and probes is available in **Supplementary Table S4**. Genes were normalized to 18s ribosomal RNA (Applied Biosystems, 4319413E) or the housekeeping genes *36B4* or *UBC* using the ΔCt method.

### Flow cytometry

Prior to collection, cells were treated with 5 µM of the zinc fluorophore Zinpyr-1 in PBS for 30 minutes at 37 °C. Organoids and Huh-7 cells were next treated with collagenase to generate single cell suspensions necessary for flow analysis. Cells were stained with the viability dye FVS700 (Becton Dickinson) and examined using the BD Bioscience LSR Fortessa Cell Analyzer or the Miltenyi Biotec MACSQuant Analyser 10. To examine individual PBMC populations, PBMCs were treated with FcR blocking reagent (Miltenyi Biotec) then labeled with the following panel: BUV395 CD3, PE-Cy7 CD56, BV711 CD14, APC-Cy7 CD19 and PE-CF594 CD11c. Flow cytometry data was analyzed using FlowJo v10.8.1.

### Statistical analysis, bioinformatics and visualization

Data was analyzed and figures generated in GraphPad Prism Version 8. Statistical tests used were performed based on the normality of the data (parametric versus non-parametric) and are indicated in figure legends along with the number of biological replicates. All *in vitro* experimentation was performed using a minimum of 2 experimental replicates and 2 technical replicates, and sample sizes have been included in figure legends. Functional annotation was performed using ConsensusPath-DB (43) and Gene Set Enrichment Analysis (GSEA) Software (44). Over-representation analysis was used to define biological processes and pathways associated with up-regulated (>1.5x) gene sets. Gene expression heat maps were generated using Morpheus software from the Broad Institute (https://software.broadinstitute.org/morpheus).

## Results

### Identification of zinc-stimulated genes by transcriptomic analyses

To identify genes that are consistently and robustly induced by zinc, we searched for publicly available transcriptomic datasets that performed zinc treatments. Of ten studies identified, eleven zinc-treatment datasets were suitable for statistical analysis (**Table 1**). Analyzed transcriptomes were heterogeneous with regard to microarray/sequencing technology used, cell type, zinc treatment (zinc salts and zinc nanoparticles), zinc concentration (25-250 µM) and treatment duration (4–24 h). As a result, the number of significantly up-regulated (≥1.5-fold induction, p<0.05) ZSGs ranged from <10 to >1000 (full list in **Supplementary File 1**).

**Table 1:** Datasets containing zinc-stimulated transcriptomes.

Dataset	Cell type	Zinc treatment	Treatment duration	# ZSGs identified	Reference
GSE76510	Caco-2 colorectal adenocarcinoma cells	150 μM ZnCl_2_	24 h	224	(20)
GSE152703^a^	MCF7 mammary adenocarcinoma cells	100 μM ZnSO_4_	6 h	0	(21)
GSE6960	A549 lung adenocarcinoma cells	25 μM ZnOAc_2_ + 10 μM PCI-5002	4 h	685	(19)
N/A	A549 lung adenocarcinoma cells	250 μM ZnCl_2_	6 h	1453	(32)
GSE2111	Primary bronchial epithelial cells	50 μM ZnSO_4_	4 h	8	(22)
GSE60159	Primary hepatic stellate cells	185 μM ZnSO_4_	24 h	328	(23)
GSE25167 ^b^	HaCat immortalized keratinocyte cells	100 μM ZnCl_2_	4 h	46	(24)
	Sk Mel-28 melanoma cells				
GSE39316 + GSE39330	Monocyte derived macrophages	122 μM ZnO nanoparticles	6 h and 24 h	139	(33)
	Monocyte derived dendritic cells			10	
	Jurkat T cell leukemia			60	
GSE2964	Ramos B cell lymphoma	50 μM ZnOAc_2_	4 h	6	(25)
GSE99435	PMA differentiated THP-1 cells	98 μM ZnO nanoparticles	4 h	26	(26)

a Removed from analysis due to insufficient statistical power to identify ZSGs.

b HaCat and Sk Mel-28 cell lines examined together to increase statistical power.

**Figure 1:**
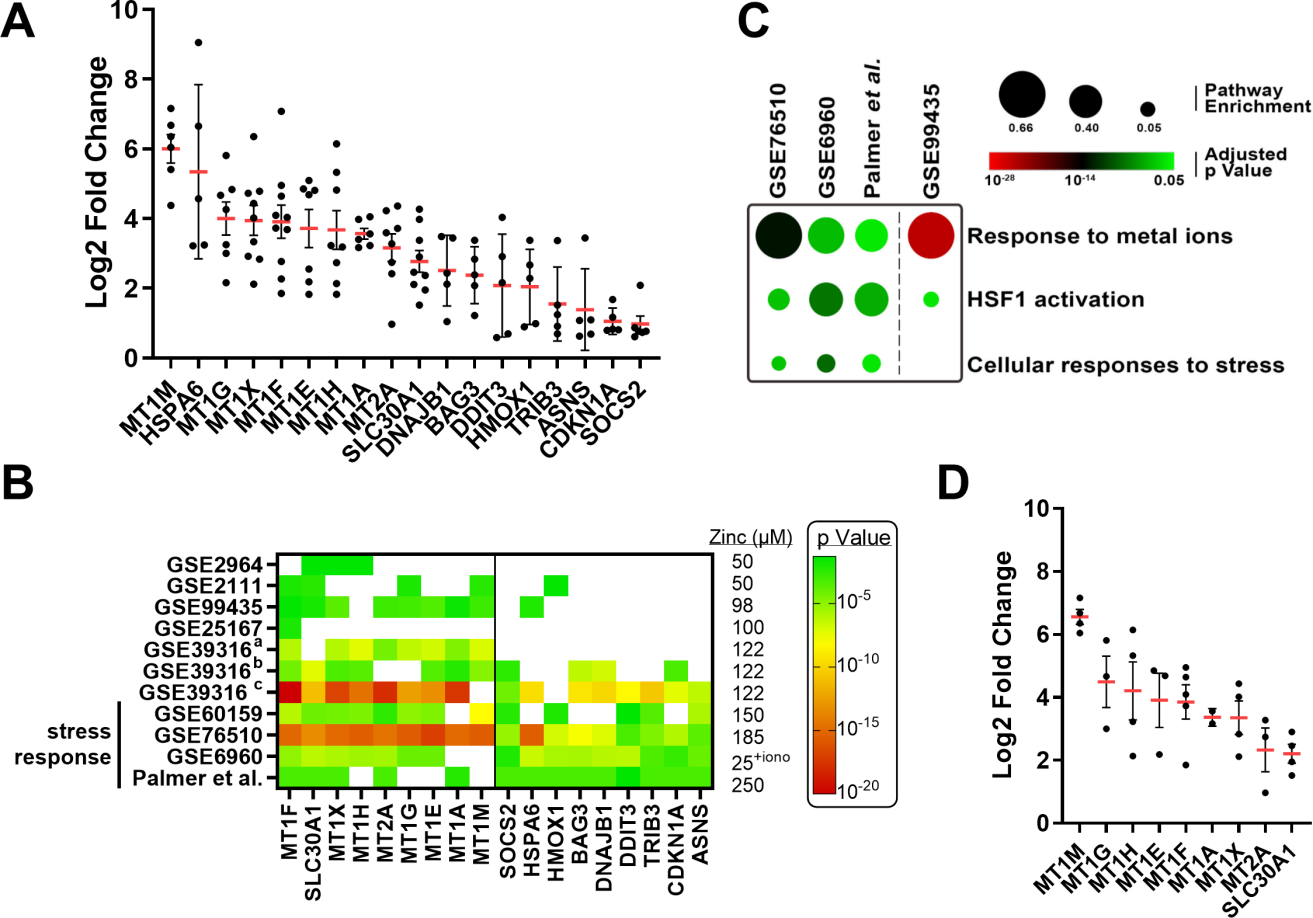
Identification of zinc-stimulated transcripts. Zinc treated datasets were queried to identify significantly up-regulated (≥1.5x) transcripts following zinc treatment. **(A)** 18 transcripts were significantly up-regulated following zinc treatment in ≥5/11 datasets. Only significant data points are shown. **(B)** Heat map representation of significantly up-regulated ZSGs among select datasets highlighting the stress response genes identified in datasets using high zinc concentrations or zinc ionophores. **(C)** Pathway enrichment of select datasets demonstrating induction of cellular stress response. **(D)** Strong induction of remaining ZSGs in the absence of datasets that elicit a stress response. HSF1, heat shock factor protein 1; iono, ionophore.

Significantly up-regulated ZSGs identified in ≥5/11 datasets were first chosen to generate a preliminary transcriptomic zinc signature (**Figure 1A**). A total of 18 transcripts were identified, dominated by genes belonging to the metallothionein (MT) gene family. In addition, a set of genes responsible for DNA damage and stress responses (*e.g. HSPA6, DDIT3)* were up-regulated suggesting that some zinc treatment experiments were eliciting significant cell stress. Indeed, when examining ZSG induction among all datasets, only those experiments using high zinc concentrations above 150 µM (*e.g.* Palmer et al. (32), GSE60159 (23)) or zinc ionophores (GSE6960 (19)) stimulated a transcriptomic stress response (**Figure 1B**). Dataset-specific transcriptional responses were confirmed using functional annotation performed using ConsensusPath-DB (43). Cell stress pathways were enriched among datasets using high zinc concentrations, whereas the GSE99435 transcriptome was enriched for metal ion response pathways, with minimal stress pathway enrichment (**Figure 1C**). As serum zinc rarely climbs above 20 µM *in vivo (*45), it is unlikely to stimulate cell stress responses identified here. Consequently, all cell stress related transcripts were removed from the preliminary zinc signature. Even in the absence of datasets eliciting zinc-mediated cell stress, the nine remaining ZSGs remained highly up-regulated (**Figure 1D**).

We next examined ZSG promoters using the online tool PScan to identify over-represented transcription factor binding motifs that may indicate up-regulation by zinc. We input 4 lists of ZSGs including the nine gene zinc signature, as well as lists representing ZSGs present in ≥5 datasets (n=18), ≥4 datasets (n=57) and ≥3 datasets (n=119). The binding motif of metal transcription factor 1 (MTF-1) was significantly enriched in all gene lists, possessing binding sites in every promoter sequence within the nine gene signature (**Supplementary Table S5**). MTF-1 is directly activated by zinc and is a major driver of MT expression, as demonstrated by knockdown studies (21).

**Figure 2:**
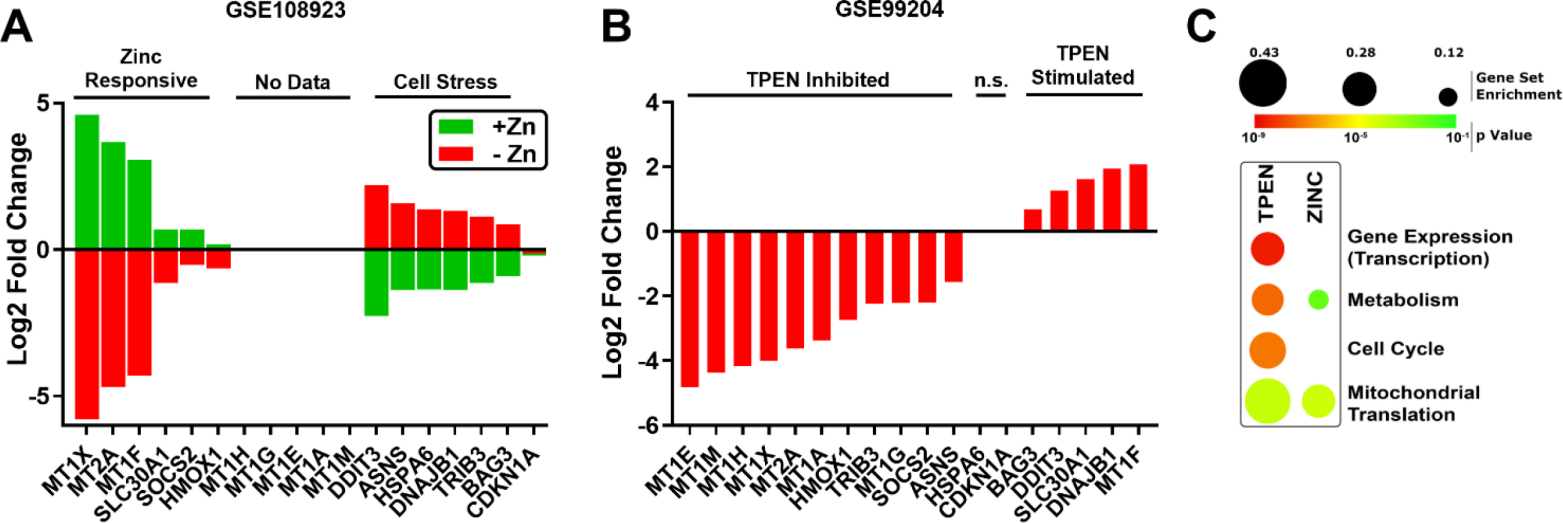
Zinc sequestration modulates zinc signature genes and homeostatic pathways. Two zinc depletion datasets were examined to determine if an absence of zinc reciprocally down-regulates the 18 zinc-stimulated transcripts. **(A)** TPEN and **(B)** S100A12 resin-based zinc removal significantly reduced overlapping but unique sets of ZSGs. **(C)** Functional annotation of cellular processes disrupted by the different methods of zinc depletion. n.s., not significant; TPEN, N,N,N’N’-tetrakis (–)[2-pyridylmethyl]-ethylenediamine; Zn, zinc.

To verify the nine gene transcriptional zinc signature following zinc depletion, we examined ZSG expression in two datasets that used different methods of zinc removal: S100A12 resin-based zinc depletion (GSE108923) (27) or TPEN-mediated ion chelation (GSE99204) (28) (full list of DEGs in **Supplementary File 2**). Confirming our hypothesis, all measured zinc signature genes were down-regulated in response to S100A12 resin-based zinc sequestration in HEK293T cells and were stimulated following the addition of zinc (**Figure 2A**). In TPEN treated human keratinocytes, zinc signature gene transcripts were significantly reduced except for *MT1F* and *SLC30A1* which were up-regulated (**Figure 2B**). In both datasets, cell stress marker expression (*e.g., DDIT3*, *DNAJB1*) was increased by zinc removal, suggesting that they are indeed not true ZSGs.

Functional annotation of transcriptomes was carried out to better understand the effect of zinc-deprivation in these datasets. S100A12-mediated zinc removal is highly specific (27), and resulted in minimal disruption of cell processes based on transcriptional responses. On the other hand, TPEN ion chelation, which is not zinc specific (46, 47), inhibited numerous homeostatic pathways including transcription, translation, metabolism and cell cycle (**Figure 2C**).

### Confirmation of zinc stimulated gene modulation in liver and immune cell models

**Figure 3:**
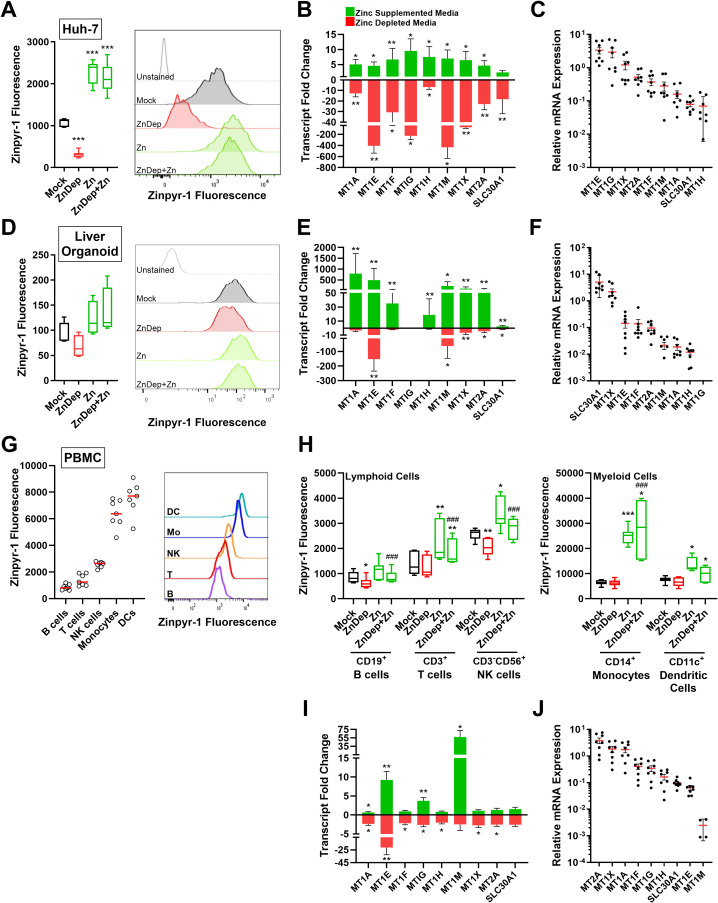
*In vitro* validation of zinc stimulated gene responsiveness to zinc depletion and zinc treatment. Huh-7 cells **(A-C)**, liver organoids **(D-F)** and PBMCs **(G-J)** were treated with control or zinc depleted media ± 50 µM ZnSO_4_ and intracellular zinc status was measured by flow cytometry and PCR. **(A)** Huh-7 cells (n=9) and **(D)** liver organoid (n=4) intracellular zinc as measured by Zinpyr-1 fluorescence. **(B)** Huh-7 cell (n=8) and **(E)** liver organoid ZSG expression as measured by qPCR (n=8). **(C)** Untreated Huh-7 cell (n=8) and **(F)** liver organoid (n=8) ZSG expression from highest to lowest. **(G)** PBMC intracellular zinc fluorescence as measured by Zinpyr-1 (n=7). **(H)** Intracellular zinc fluorescence of PBMC populations following zinc depletion and zinc treatment (n=7). **(I)** PBMC ZSG expression following zinc depletion and zinc treatment (n=8). PBMC ZSG expression from highest to lowest(n=4-8). **(B, E, I)** Wilcoxon matched pairs signed rank test versus mock, **(A, D, H)** one way ANOVA with repeated measures, * p<0.05, ** p<0.01, ***p<0.001, * versus mock treatment, # versus Zinc depleted media. DC, dendritic cell; NK, natural killer; Mo, monocyte; Zn, zinc; ZnDep, zinc depleted media.

In order to resolve the inconsistent modulation of ZSGs (GSE99204) and absence of MT expression data (GSE108923) we performed zinc treatment and depletion experiments *in vitro*. To model the major hepatic cell populations, three *in vitro* models were used: Huh-7 hepatoma cells and primary liver organoid monolayers were used to model hepatocytes and peripheral blood mononuclear cells (PBMCs) to model resident and infiltrating immune cells. All cells were cultured in mock treated or S100A12 resin zinc depleted (ZnD) media for 24 h ± supplementation with 50 µM ZnSO_4_. Intracellular zinc was quantified using the zinc fluorophore Zinpyr-1 and ZSGs quantified by qPCR. Compared to untreated Huh-7 cells, zinc depletion and zinc treatment resulted in a significant reduction and increase in cellular zinc content, respectively, as measured by Zinpyr-1 fluorescence (**Figure 3A**). Consistent with cellular zinc content, all ZSGs were significantly down-regulated following zinc depletion, apart from *SLC30A1*, and significantly induced following zinc treatment (**Figure 3B**). Genes demonstrating the strongest response to zinc concentration (*e.g., MT1E*, *MT1G*) were generally highest expressed in untreated Huh-7 cells (**Figure 3C**). Liver organoid intracellular zinc was modulated by zinc depletion and treatment (**Figure 3D**, p>0.05), however to a far lesser degree than Huh-7 cells. Similarly, liver organoid ZSG expression was less affected by zinc depletion and highly sensitive to zinc treatment (**Figure 3E**). Organoid ZSG expression was also unique, with *SLC30A1* demonstrating the highest relative expression and *MT1G* below the limit of detection (**Figure 3F**).

Following zinc depletion and supplementation, PBMC populations were also examined by flow cytometry to assess intracellular zinc content (gating in **Supplementary Figure S3**). Untreated dendritic cells (DCs) and monocytes possessed larger intracellular zinc pools compared to lymphocyte populations, as demonstrated by higher Zinpyr-1 fluorescence (**Figure 3G**). Interestingly, only lymphoid cells were significantly affected by zinc depletion, showing a reduction in zinpyr-1 fluorescence (B cells and NK cells, p<0.05) (**Figure 3H**). Except for T cells, all cell populations exhibited increased zinpyr-1 fluorescence in response to zinc treatment, with monocytes demonstrating a potent zinc influx as compared to other cells. PBMC *MT1A*, *MT1E*, *MT1G* and *MT1M* were stimulated by zinc, whereas all genes except *MT1M* and *SLC30A1* were significantly reduced by zinc depleted media (**Figure 3I**). Baseline ZSG expression exhibited very low *MT1M* expression as compared to other ZSGs (**Figure 3J**), perhaps reflecting the strong induction following zinc treatment.

To assess the zinc-specific induction of the ZSGs, PBMCs were additionally treated with 50 µM metal salts containing divalent cations calcium (Ca^2+^), copper (Cu^2+^) manganese (Mn^2+^), magnesium (Mg^2+^) and iron (Fe^2+^) in addition to zinc (Zn^2+^) (**Supplementary Figure S4**). In addition to zinc, copper treatment demonstrated a significant induction of multiple ZSGs.

### The hepatic zinc signature is diminished in alcohol related liver disease

**Figure 4:**
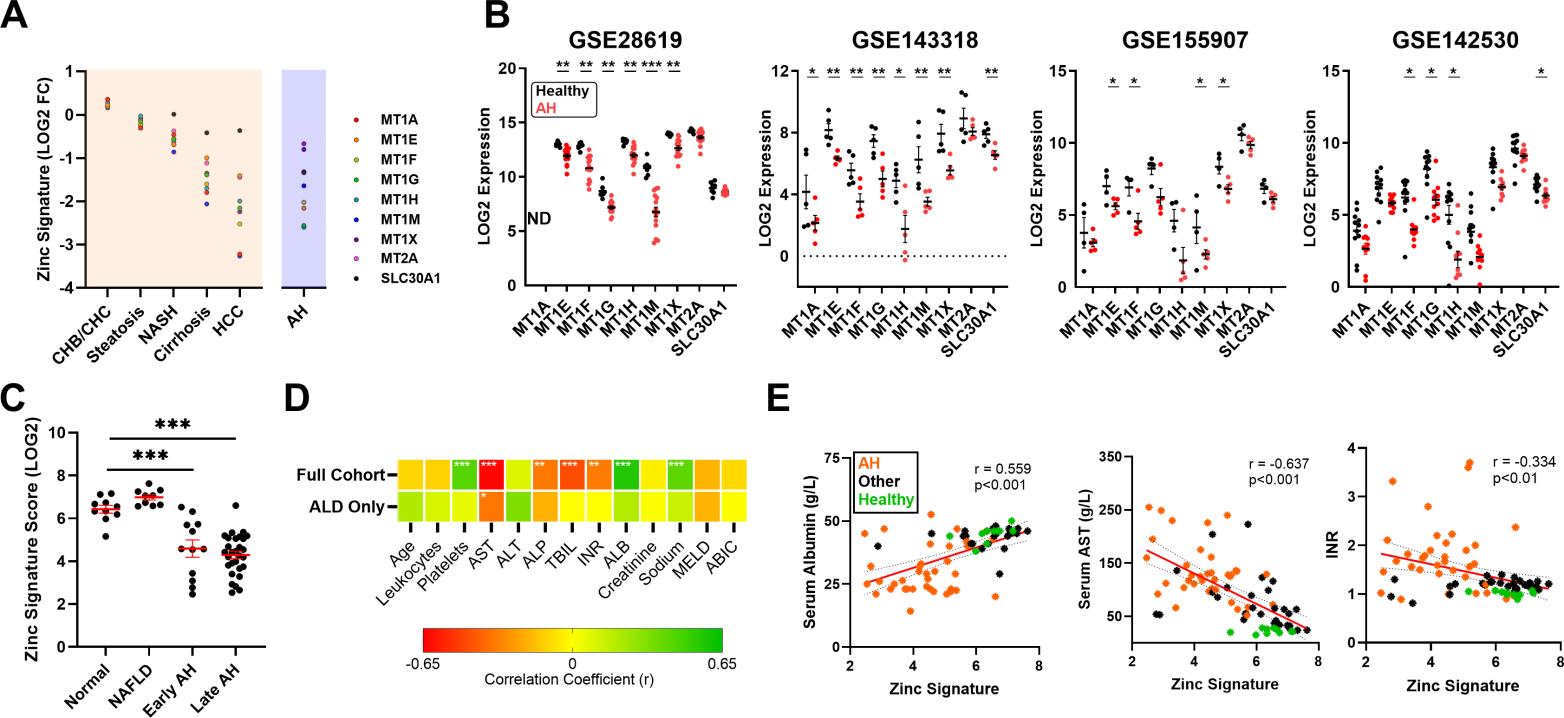
Hepatic zinc content is reduced in alcohol related liver disease and is associated with hepatocyte dysfunction. A zinc signature score was created using the average normalized expression count of the nine validated ZSGs. **(A)** LOG2 fold change (FC) of the zinc signature score across different chronic liver disease datasets to assess the degree of zinc deficiency compared to healthy tissue. **(B)** ZSG expression in healthy and AH liver tissue from datasets GSE28619 (n=22), GSE143318 (n=10), GSE155907 (n=10) and GSE142530 (n=22). **(C)** Zinc signature scores from dbGaP dataset phs003112.v1.p1 (n=79) comparing early and late alcohol-associated hepatitis to normal and MAFLD liver. **(D)** Correlation analysis using the full cohort (n=79) or patients with AH only (n=41) to assess the relationship between hepatic zinc signature scores and clinical parameters. **(E)** Scatter plots demonstrating the correlation between hepatic zinc signature scores and albumin, AST or INR. Wilcoxon signed-rank test **(B)**, One way ANOVA with repeated measures **(C)**, Pearson correlation **(D, E)**, * p<0.05, ** p<0.01, ***p<0.001. ABIC, age bilirubin INR creatinine Score; AH, alcohol-associated hepatitis; ALB, albumin; ALD, alcohol related liver disease; ALP, alkaline phosphatase; ALT, alanine aminotransferase; AST, aspartate aminotransferase; CHB, chronic hepatitis B; CHC, chronic hepatitis C; HCC, hepatocellular carcinoma; INR, international normalized ratio; MELD, model for end-stage liver disease; MAFLD, metabolic dysfunction-associated fatty liver disease; NASH, non-alcoholic steatohepatitis; ND, no data; TBIL, total bilirubin.

To compare hepatic zinc status among different chronic liver diseases, transcriptomic datasets containing data from healthy and diseased liver tissue were queried (full list in **Supplementary Table S6**). To simplify our analysis, a zinc signature score was generated using the average normalized log counts per million (CPM, RNA sequencing platforms) or log intensity (microarray platforms) of the nine validated ZSGs. All 9 ZSGs were given identical weights within the zinc signature score to facilitate the use of the zinc signature across datasets and tissues where expression of individual ZSGs differs significantly, as demonstrated in **Figures 3C, F, J**. ZSG expression data was obtained from chronic hepatitis B virus (CHB, n=3) and chronic hepatitis C virus (CHC, n=1) infected, steatosis (n=5), non-alcoholic steatohepatitis (NASH, n=5), hepatocellular carcinoma (HCC, n=4), cirrhosis (n=5) and alcohol-related liver disease (ALD, n=4) liver tissue, and compared to healthy controls available within each dataset to generate a LOG2 fold change (FC) for each gene (**Figure 4A**). While chronic viral infection and steatosis did not affect ZSG expression, ZSGs decreased in response to chronic inflammation (*e.g.*, NASH) and fibrosis, reaching a minimum in HCC tumor tissue. Among non-cancerous tissue, ALD and cirrhotic liver ZSG expression were most significantly reduced compared to healthy tissue, supporting a zinc deficient state in the liver.

To further investigate the four AH datasets outlined in **Figure 4A**, ZSG expression was examined from healthy and severe AH liver tissue transcriptomes: GSE28619 (n=7 normal, n=15 severe AH [Maddrey’s discriminant function >32]) (34), GSE143318 (n=5 normal, n=5 severe AH) (35), GSE155907 (n=4 normal, n=6 AH undergoing liver transplant) and GSE142530 (n=12 normal, n=10 severe AH) (37). Among the four datasets, all ZSGs were consistently down-regulated, with *MT1F* reaching significance in all datasets (**Figure 4B**).

We next queried an additional liver tissue dataset (accession phs003112.v1.p1 (40)) containing demographic and blood test results from the database of Genotypes and Phenotypes (dbGaP) to determine whether hepatic zinc status, as defined by the transcriptomic zinc signature score, correlates with any clinical parameters. Of the 79 liver transcriptomes contained within the dataset, 10 were classified as normal, 41 AH, 19 chronic HCV infection and 10 MAFLD. Consistent with datasets outlined in **Figure 4A**, the MAFLD zinc signature score was similar to healthy liver, whereas both acute and chronic AH demonstrated a significantly reduced zinc signature score (**Figure 4C**).

Individual zinc signature scores were next correlated against demographic information (age), blood test results (*e.g.*, leukocyte count), liver function tests (*e.g.*, AST) and predictive scores (*e.g.*, model for end-stage liver disease [MELD]). Using the entire dataset, the zinc signature score demonstrated a strong positive correlation with platelet count, albumin (ALB) and sodium, as well as a similarly strong negative correlation with AST, ALP, TBIL and INR. Among the AH patient subgroup (n=41), only the negative correlation with AST remained significant (**Figure 4D**). Scatter plots depicting correlation between the zinc signature score and albumin, AST and INR (**Figure 4E**) highlight the disparity between patients with ALD and the remainder of the cohort. Among patients with ALD, a low hepatic zinc signature score was associated with low serum albumin and low platelet count (thrombocytopenia) and prolonged clotting time (INR) suggestive of poor liver function, as well as elevated AST, a marker of liver inflammation and hepatocyte death. Correlation and regression analysis data from **Figure 4** is available in **Supplementary File 3**.

### Assessing the role of hepatic zinc in alcohol-related liver disease

**Figure 5:**
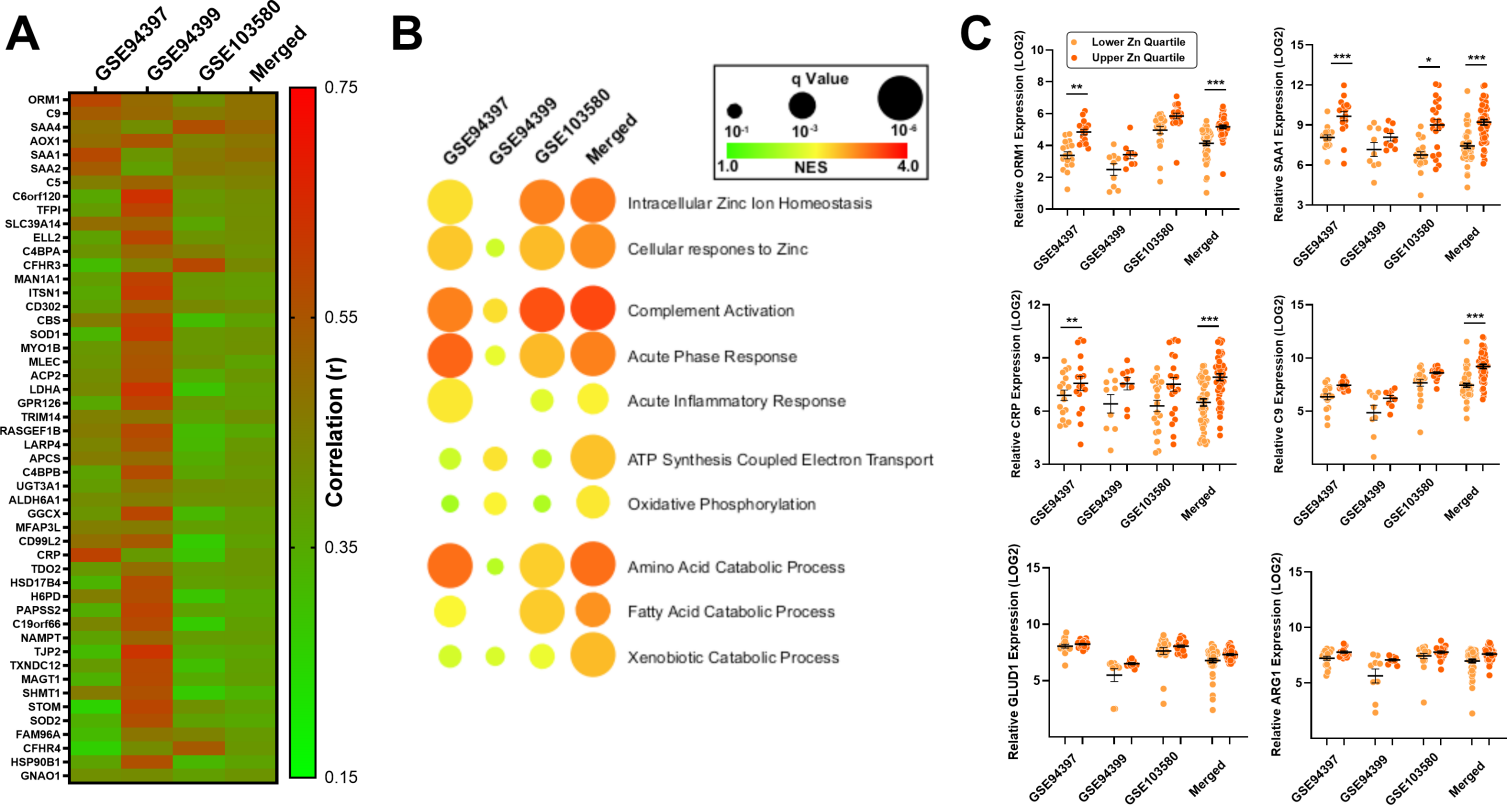
Inflammatory and metabolic pathways are associated with the hepatic zinc signature in alcohol related liver disease. AH biopsy tissue transcriptomes from datasets GSE94397 (n=71), GSE94399 (n=38) and GSE103580 (n=110) and a merged dataset (n=219) were queried to assess the role of hepatic zinc in AH pathology. **(A)** Top 50 genes among four AH datasets that correlate with the hepatic zinc signature score. **(B)** Gene set enrichment of correlation ranked lists demonstrating conserved biological processes among the four AH datasets. **(C)** Comparison of relevant acute phase response and amino acid catabolism genes among the top quartile (highest zinc signature score) and bottom quartile (lowest zinc signature score) biopsy samples. Wilcoxon signed-rank test, * p<0.05, ** p<0.01, *** p<0.001. NES, normalized enrichment score; Zn, zinc.

To determine the role of hepatic zinc in AH pathogenesis, three severe AH datasets of liver biopsy transcriptomes were queried: GSE94397 (n=71), GSE94399 (n=38) and GSE103580 (n=110). In addition, a merged, batch-corrected dataset was generated from all three datasets containing 219 samples. A hepatic zinc signature score was generated for every patient sample and was and was subsequently correlated against every other gene across all four datasets. **Figure 5A** demonstrates the top 50 positively correlated genes across all four datasets (**Supplementary File 4**). The zinc signature score was found to correlate positively with multiple genes belonging to members of the acute phase response to infection, including complement (*C5*, *C9*), serum amyloid proteins (*SAA1*, *SAA2*), and C-reactive protein (*CRP*). Gene set enrichment analysis of resulting correlation coefficient ranked lists (**Figure 5B**) demonstrated that the zinc signature score was associated with zinc homeostasis and response, as well as acute inflammatory responses including complement activation and the acute phase response. Interestingly, enrichment of bioenergetic and metabolic responses were also identified including ATP synthesis and oxidative phosphorylation, as well as amino acid/fatty acid catabolism. Samples from each dataset were next grouped into quartiles based on their zinc signature scores and compared (**Figure 5C**). The top quartile, representing patients with the highest zinc signature scores, were compared to the bottom quartile with the lowest zinc signature scores. Acute phase response genes orosomucoid 1 (*ORM1)*, serum amyloid A1 (*SAA1)*, C-reactive protein (*CRP)* and complement component 9 (*C9)* were consistently higher among patients with higher zinc signature scores. Similarly, protein catabolism genes such as glutamate dehydrogenase (*GLUD1)* and arginase 1 (*ARG1*) responsible for catabolism of glucogenic amino acids glutamic acid and arginine were consistently higher in all datasets, however no significant differences were observed.

### Examining the effect of zinc deficiency on the acute phase response

**Figure 6:**
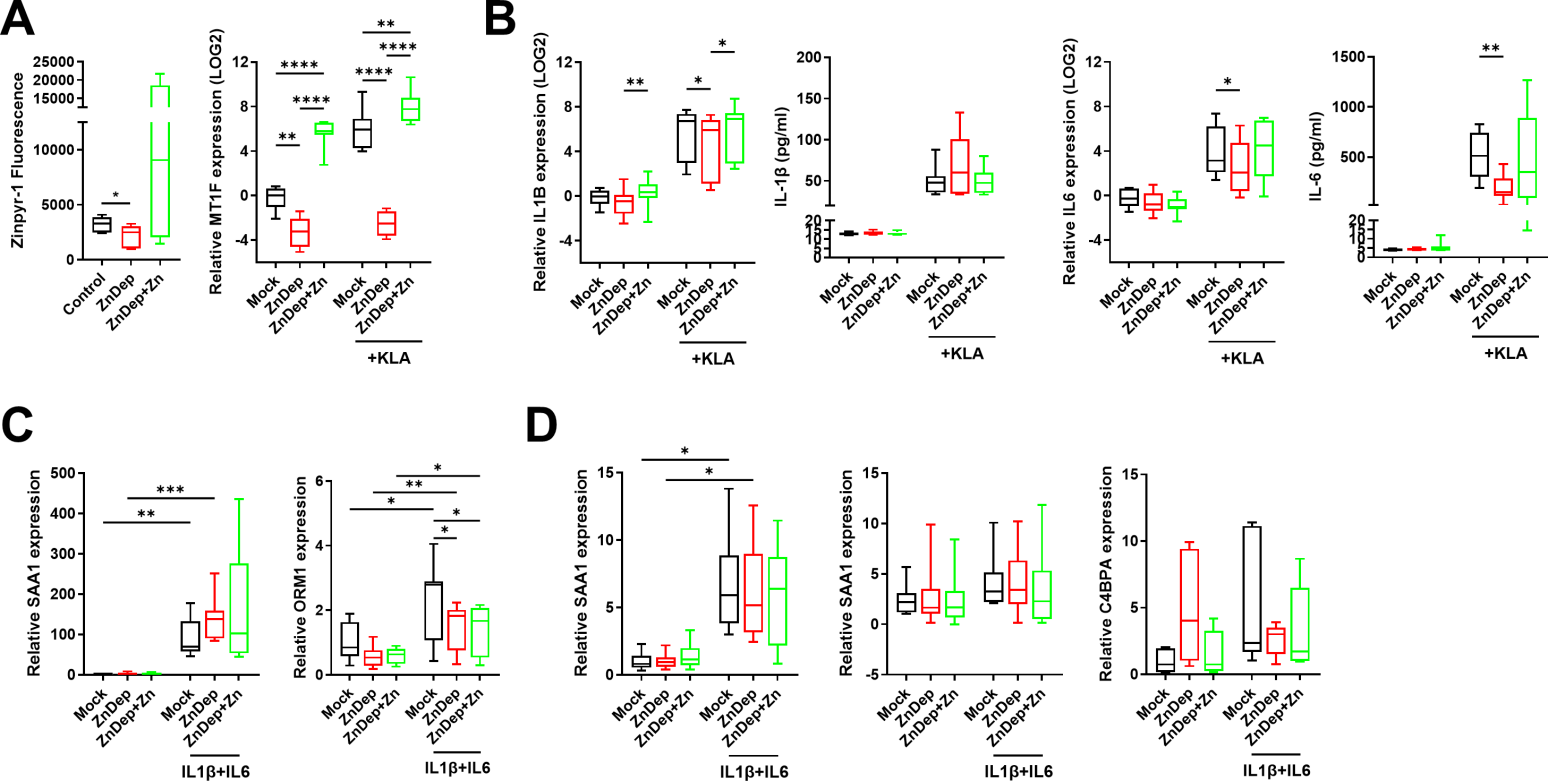
Zinc deficiency reduces the macrophage inflammatory response without affecting hepatocytes. Monocyte-derived macrophages were cultured in zinc-depleted and supplemented media followed by treatment with KLA to assess the induction of inflammatory cytokines. **(A)** Macrophage zinc content as measured by Zinpyr-1 fluorescence (n=7) and *MT1F* gene expression (n=9). **(B)** Macrophage *IL1B* and *IL6* gene expression and secretion into media following treatment with KLA (n=9). **(C)** Huh-7 induction of acute phase reactant genes *SAA1* and *C9* following treatment with IL-1β and IL-6 for 6 h (n=9). **(D)** Liver organoid monolayer induction of acute phase reactant genes *SAA1* and *C4BPA* following treatment with IL-1β and IL-6 for 6 h (n=9). Quantification of SAA1 in culture media was also performed by ELISA (n=8). One way ANOVA with repeated measures, * p<0.05, ** p<0.01, ***p<0.001, ****p<0.0001. KLA, Kdo2-Lipid A; Zn, zinc; ZnDep, Zinc depleted.

Zinc influx into the liver occurs rapidly during the acute phase response, so we sought to determine whether hepatic zinc is truly a requirement for the production of acute phase reactants. The acute phase response is a response to infection initiated in the liver following hepatocyte stimulation by inflammatory mediators such as IL-6, IL-1β and TNF. Because macrophages are the primary mediators of the hepatic inflammatory response, we first sought to examine macrophage IL-6 and IL-1β production in response to zinc depletion. Monocyte-derived macrophages were cultured in mock, ZnD or ZnD + 50 μM ZnSO_4_ media overnight, then simulated with 100 ng/ml Kdo2-Lipid A (KLA), the immunoreactive component of lipopolysaccharide (LPS) for 6 h. Macrophage intracellular zinc and *MT1F* expression were significantly reduced by zinc depleted media supporting a reduction in intracellular zinc (**Figure 6A**). KLA-induced *IL6* and *IL1B* expression were significantly reduced following zinc depletion and restored with the addition of ZnSO_4_ (**Figure 6B**). Secreted IL-6 was reduced in response to zinc depletion, whereas IL-1β remained unaffected.

IL-1β and IL-6 (20 ng/ml each) were next used to stimulate the acute phase response in both Huh-7 cells (**Figure 6C**) and liver organoid monolayers (**Figure 6D**). Both Huh-7 *SAA1* and *ORM1* expression were increased following 6 h IL-1β and IL-6 treatment, however only *ORM1* was affected by cellular zinc status, demonstrating a reduction in expression in zinc depleted samples, even following the addition of zinc. SAA1 protein secretion was measured in Huh-7 culture media following cytokine stimulation but could not be detected. Consistent with Huh-7 findings, SAA1 transcript expression and protein secretion remained unaffected by zinc concentration in the organoid model. *ORM1* could not be detected in organoid cultures, however the acute phase reactant *C4BPA* was similarly unaffected by zinc. Together, these data indicate that zinc deficiency in AH may dampen the initiation of the acute phase response by limiting the expression of inflammatory mediators in the liver.

## Discussion

While the broad clinical symptoms of zinc deficiency have been well described in humans, its unique effects on organs and systems have been difficult to establish due to the limitations of clinical research: Complex clinical cases with numerous independent variables, patient co-morbidities, and limited access to tissue to name a few. To help overcome these constraints, we have developed and validated a nine-gene zinc signature to enable the estimation of zinc content within tissue that is supported by human (48) and murine zinc supplementation (49, 50) and zinc restriction (51) studies. The findings of this work can therefore be divided into two themes that will be discussed herein: 1) The use of contemporary methodologies to generate and validate a transcriptomic zinc signature to quantify tissue zinc, and 2) combining primary cell culture models and novel zinc depletion methodologies to demonstrate how zinc deficiency affects the hepatic acute phase response to infection. We have used zinc-deficient ALD liver transcriptomes to investigate zinc deficiency in tissue, however the proposed zinc signature can likely be used to interrogate zinc status in diverse tissues and disease states.

Established methods to investigate the role of intracellular zinc on signaling pathways have largely relied on zinc chelators such as TPEN and cancer cell lines, whose results are inconsistent with our findings (52). While TPEN is used to chelate zinc, it has been used in cell culture studies to sequester copper (53), calcium (46) and iron (47) in addition to zinc. TPEN is cell permeable, and can thus displace metals that occupy enzymatic, nucleic acid binding, and other roles within the cell (54), poorly representing the zinc deficient state as it occurs *in vivo*. Our method of zinc depletion used a resin bound with S100A12 (27), an antimicrobial peptide that can sequesters Zn^2+^ ions with nanomolar affinity (55). S100A12 resin possesses selectivity for Zn^2+^ removal (99% depletion) without affecting the concentrations of calcium (Ca^2+^), magnesium(Mg^2+^), iron (Fe^2+^), cobalt (Co^2+^) or copper (Cu^2+^) (27).

Cancer cell lines have also been regularly used to examine zinc biology, however zinc is commonly dysregulated in cancer where it can contribute to carcinogenesis and disease progression (56). Cancer cell lines are no exception. As compared to PBMCs and primary liver organoid monolayers, Huh-7 cells were significantly more sensitive to changes in intracellular zinc content, as measured by Zinpyr-1 fluorescence (**Figures 3A, D, H**). These data are consistent with published data (57) and suggest that cancer cell lines are not an optimal model to study physiological responses to zinc. Indeed, we observed a strong transcriptomic response to KLA, but no measurable secretion of SAA1 in Huh-7 cells, reflecting their poor suitability for the study of the acute phase response.

The nine gene zinc signature identified herein is a significant advantage over single-gene biomarkers, whose expression (or lack thereof) can differ significantly between cell types and tissues (**Figure 3**). While a variety of zinc biomarkers have been proposed such as superoxide dismutase and fatty acid desaturase (58), 1) they are not superior to serum zinc, 2) their expression is affected by other inflammatory/metabolic stimuli and 3) they do not reflect tissue-specific zinc status. By using the zinc signature to quantify hepatic zinc content, we identified significant zinc deficiency among AH liver biopsies (**Figures 4A–C**), consistent with clinical findings demonstrating serum zinc deficiency in up to 85% of patients with AH (59). This remained significant irrespective of tissue collection (biopsy vs explant) and duration of disease (acute vs chronic AH), substantiating the strong association between alcohol and reduced hepatic zinc content. We further identified an inverse relationship between the hepatic zinc signature and liver injury (**Figures 4D, E**); a finding supported by animal models (60, 61) as well as clinical findings in humans (16). Indeed, a 2018 study by Vatsalya et al. demonstrated that serum zinc was inversely associated with the AST: ALT ratio and C-reactive protein (CRP), a systemic marker of inflammation among alcohol-dependent patients (16).

Using large datasets of AH liver tissue, we were next able to identify a significant association between the hepatic zinc signature and expression of acute phase reactants such as *CRP*, SAA genes (*SAA1, SAA2, SAA4*) and complement genes (*C5, C9, C4BPA, C4BPB*) (**Figure 5**). The acute phase response is initiated by macrophages in response to infection, by secreting inflammatory cytokines such as IL-6, TNF and IL-1β. These inflammatory cytokines act on hepatocytes to stimulate a rapid influx of zinc into hepatocytes followed by the production of acute phase reactants (62, 63). Zinc movement from blood into the liver is a well-established element of the acute phase response, but whether it is *indispensably required* to initiate the acute phase response remains uncertain. Using zinc depleted media, we demonstrated that macrophage response to LPS was sensitive to zinc depletion, but hepatocyte induction of acute phase reactants was likely not. Similar to hepatocytes, acute zinc influx occurs in monocytes and macrophages following stimulation with LPS and is necessary for the efficient production of inflammatory cytokines such as TNF and IL-1β (64). The use of zinc-depleted media in this study demonstrates that macrophages zinc influx can promote the production of acute phase reactants, particularly the production of IL-6. As zinc is a potent inhibitor of intracellular phosphatases (65), this transient zinc influx may promote NF-kB signaling by preventing the dephosphorylation of signaling intermediates such as mitogen-activated protein kinase (MAPK) and IκB kinases (66, 67).

Conversely, hepatocytes were able to maintain production of acute phase reactants in zinc depleted media, suggesting that zinc influx is either not essential for initiation of the acute phase response, or that zinc depletion was limited in our model and cultured hepatocytes contain an intracellular zinc store that can be utilized during acute zinc depletion. It is therefore possible that chronic zinc deficiency *in vivo* may limit hepatocyte production of acute phase reactants due to a more pronounced hepatic zinc deficiency, and merits further examination.

A recent study examining zinc supplementation in combination with pentoxifylline (PTX) and the IL-1R antagonist, anakinra found no improvement in alcoholic hepatitis patient outcomes as compared to standard of care methylprednisolone (68). Incidence of infection was similar among both groups, however fungal infections were reduced among patients treated with anakinra, PTX and zinc. These data are promising but it is difficult to decipher the role of zinc in this context. Additional clinical studies will be necessary to define the effect of different zinc supplementation modalities on hepatic zinc stores and incidence of infection. Albumin infusion, for example, may provide significant benefit to ALD patients when administered with zinc to increase systemic zinc concentration while leveraging the intrinsic anti-inflammatory and antioxidant effects of albumin and zinc (69). Looking forward, the broad aim of zinc supplementation would be to reduce chronic hepatic inflammation and subsequent tissue damage and fibrosis while strengthening the acute response to infection. While hepatic acute phase reactants are produced in response to an inflammatory cytokine stimulus, the short-term benefit of fighting off infection likely outweighs the potential for additional tissue damage.

There are some limitations of this study that must be considered. In addition to zinc, other divalent ions such as copper can increase the expression of the zinc signature metallothionein genes (**Supplementary Figure S2**) (70). Copper status must therefore be considered when using the zinc signature score. Nonetheless, alcoholic hepatitis, other inflammatory and fibrotic liver diseases are largely associated with an increase in systemic copper, suggesting that copper likely does not play a role in zinc signature gene down-regulation that we observed in AH patients (**Figure 4**). An additional limitation of this study is the lack of *in vivo* models. Indeed, zinc deficiency has been examined using murine sepsis models, however these data were complicated by significant immunodeficiencies associated with a zinc restrictive diet (52, 71, 72). Zinc deficient mice exhibited increased bacterial burden and mortality, increased hepatic inflammation and hepatocyte apoptosis, in addition to significant increases in *IL6*, *TNF* and *IL1B* and the acute phase protein SAA1 (52, 71, 72). Consequently, the specific role of hepatic zinc deficiency in this context becomes difficult to determine as it is confounded by systemic immunodeficiency and increased bacterial burden.

## Conclusion

In summary, we have developed a gene signature that can be used to estimate zinc content within tissue. We have identified a zinc deficient gene signature in the livers of patients with AH, consistent with studies that have examined zinc in serum (16, 17). Further, we have detected a strong relationship between zinc status in the liver, and induction of the acute phase response with *in vitro* findings suggesting that zinc deficiency dampens macrophage responses to LPS. Together, these data suggest that zinc deficiency may limit the efficacy of the acute phase response, dampening antibacterial immunity, and contributing to poor outcomes in patients with AH.

## Data Availability

Bioinformatics pipelines used as part of this manuscript can be found at https://github.com/ScottReadWIMR/ZincALD. Further data supporting the conclusions of this article will be made available by the authors upon reasonable request.
