# Supplementary material for: Hepatic zinc deficiency dampens the acute phase response in patients with alcohol-associated hepatitis
Source: Front Immunol. 2026 Feb 26;16:1642163. doi: 10.3389/fimmu.2025.1642163 (PMC12979129; doi:10.3389/fimmu.2025.1642163)
Supplement: Supplementary file 1 [file DataSheet1.docx]

**Supplementary Information**

**Supplementary Methods**

**
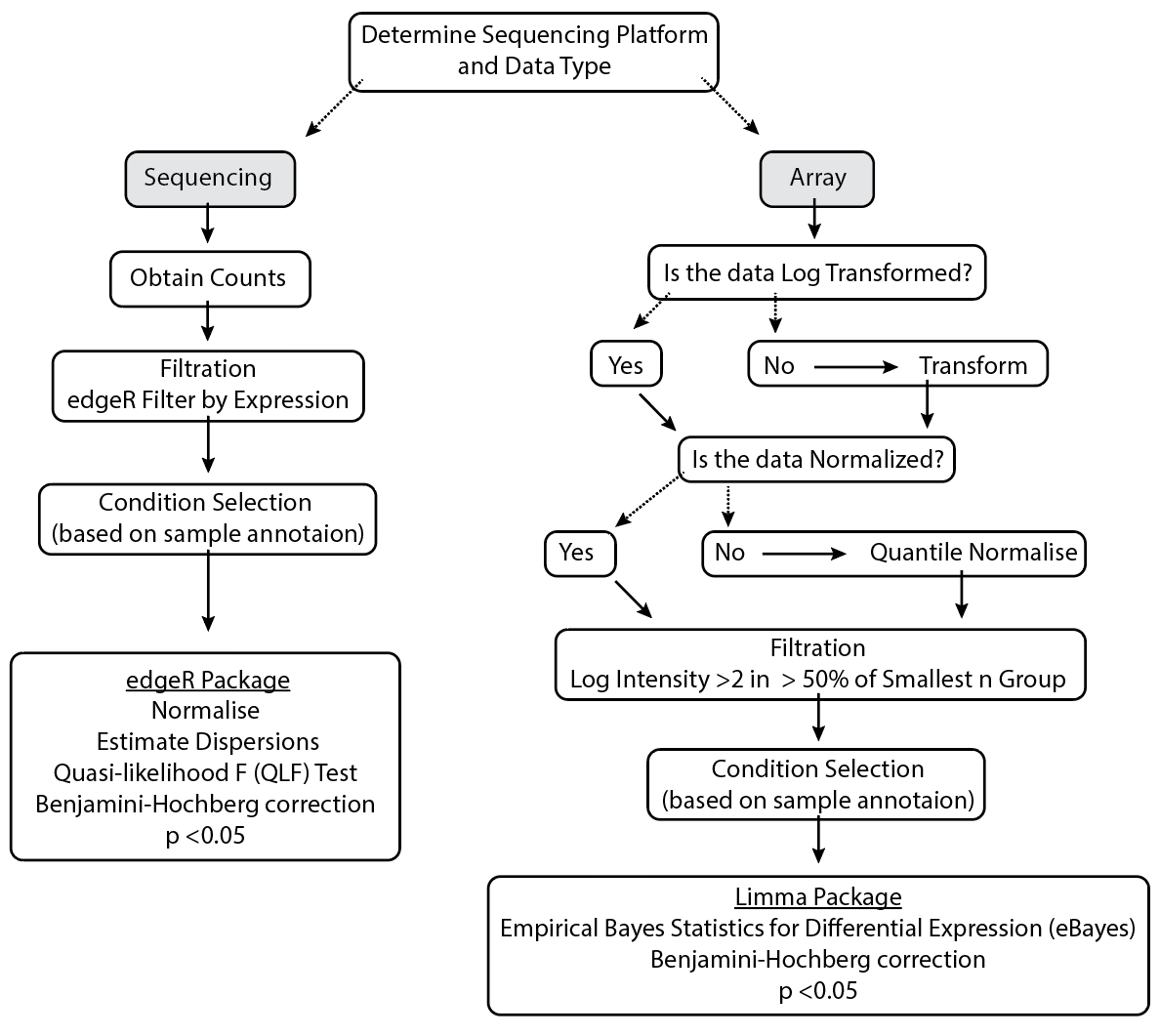
**

**Figure S1. Dataset processing tree.** RNA sequencing count-based data and array-based gene expression data processing pipelines to identify zinc-stimulated gene expression (Figures1 & 2) and liver tissue gene expression (Figures 4 & 5).


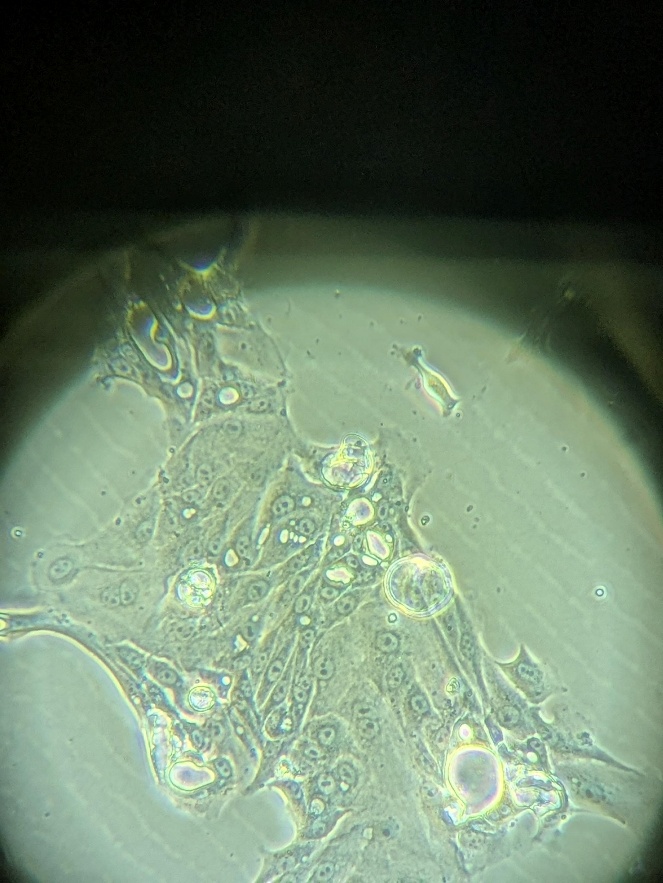

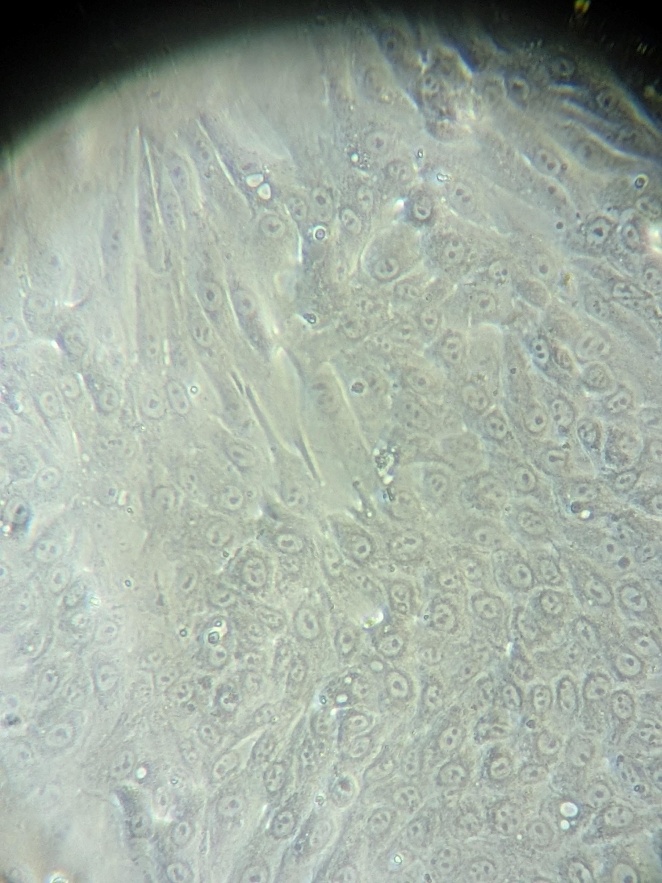

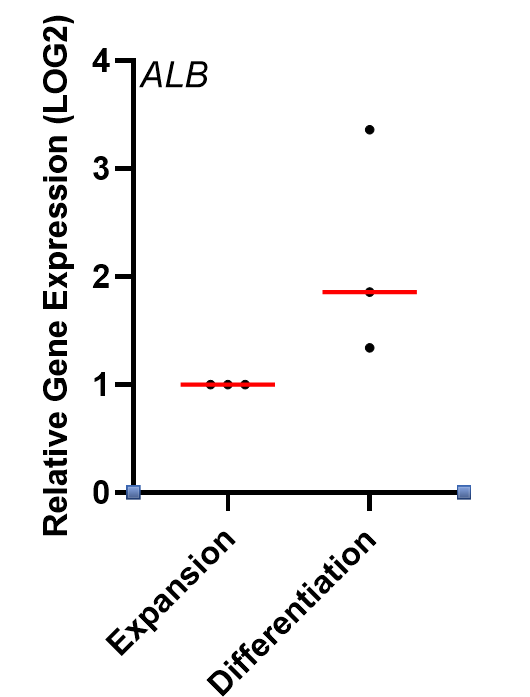


**C**

**B**

**A**

**Figure S2. Liver organoid expansion and differentiation.** Bright field images of liver organoid monolayer cultures during expansion (A) and differentiation (B), noting the presence of binucleated hepatocytes (arrow). Relative gene expression of albumin (*ALB*) following hepatocyte differentiation (n=3).

***Table S1. Zinc-stimulated gene expression among zinc treated datasets (Log transformed)***

|  | **Caco-2 cells** | **Ramos B cells** | **Primary bronchial epithelial cells** | **A549 cells** | **Primary hepatic stellate cell** | **THP-1 cells** | **HaCat keratinocytes** | **MDDC** | **MDM** | **Jurkat** | **A549** |  |  |
| --- | --- | --- | --- | --- | --- | --- | --- | --- | --- | --- | --- | --- | --- |
| **Gene** | **GSE76510** | **GSE2964** | **GSE2111** | **GSE6960** | **GSE60159** | **GSE99435** | **GSE25167** | **GSE39316 GSE39330** | **GSE39316 GSE39330** | **GSE39316 GSE39330** | **Palmer et al.** | **Up-regulated (p<0.05)** | **Expression Data Available** |
| **SLC30A1** | 2.370 | 2.906 | 2.466 | 3.979 | 1.952 | 2.112 | 1.443 | **NP** | 1.524 | 4.273 | 3.377 | 9 | 10 |
| **MT1X** | 4.790 | 2.838 | 0.304 | 2.920 | 2.117 | 4.731 | 2.127 | 4.436 | 4.018 | 3.335 | 6.357 | 9 | 11 |
| **MT1G** | 4.835 | 1.201 | 5.815 | 2.161 | 3.004 | 4.264 | 2.243 | 4.674 | **NP** | 3.253 | **NP** | 7 | 9 |
| **MT1F** | 3.684 | 2.870 | 1.852 | 2.772 | 4.954 | 2.271 | 3.729 | 4.099 | 4.647 | 4.039 | 7.081 | 10 | 11 |
| **MT1H** | 4.817 | 3.229 | 0.057 | 2.738 | 2.140 | 1.825 | 2.046 | 5.326 | 6.144 | 3.190 | **NP** | 8 | 10 |
| **MT1M** | 4.379 | -0.330 | 6.356 | 0.281 | 7.159 | 5.410 | 2.256 | 6.044 | 6.680 | **NP** | **NP** | 6 | 9 |
| **MT1E** | 4.811 | 1.060 | 0.472 | 1.825 | 2.189 | 5.099 | 2.462 | 4.685 | 4.854 | 2.557 | **NP** | 7 | 10 |
| **MT2A** | 4.226 | 3.048 | 0.028 | 2.420 | 0.974 | 3.596 | 2.279 | 3.283 | 2.742 | 3.724 | 4.366 | 8 | 11 |
| **SOCS2** | 0.771 | 1.262 | 0.893 | 0.773 | 0.872 | 0.087 | 0.226 | **NP** | 0.739 | 0.616 | 2.087 | 6 | 10 |
| **MT1A** | 4.057 | **N/A** | **N/A** | **N/A** | **N/A** | 3.229 | 2.070 | 3.562 | 3.172 | 3.403 | 3.978 | 6 | 7 |
| **HSPA6** | 3.223 | -1.424 | -0.261 | 6.654 | 0.091 | 3.247 | 0.164 | **NP** | **NP** | 4.569 | 9.057 | 5 | 9 |
| **HMOX1** | 0.988 | 0.290 | 2.287 | 2.667 | 0.897 | 1.420 | 0.302 | **NP** | **NP** | **NP** | 3.375 | 5 | 8 |
| **BAG3** | 1.218 | 0.105 | 0.004 | 2.386 | 0.100 | 0.139 | 0.063 | **NP** | 2.080 | 3.382 | 2.834 | 5 | 10 |
| **DNAJB1** | 1.050 | 0.071 | **N/A** | 3.491 | 0.415 | 0.946 | -0.093 | **NP** | 2.124 | 3.444 | 2.440 | 5 | 9 |
| **DDIT3** | 0.697 | 0.048 | -0.365 | 4.038 | 0.587 | 0.055 | -0.049 | **NP** | **NP** | 2.915 | 2.161 | 5 | 9 |
| **TRIB3** | 0.699 | 0.224 | -0.569 | 1.247 | 1.520 | -0.621 | 0.169 | **NP** | **NP** | 3.369 | 0.916 | 5 | 9 |
| **CDKN1A** | 0.833 | -0.707 | -0.650 | 0.811 | 0.275 | -0.866 | 0.081 | **NP** | 1.173 | 1.679 | 0.793 | 5 | 10 |
| **ASNS** | 0.631 | 0.027 | -0.301 | 1.098 | 3.446 | -0.331 | -0.193 | **NP** | **NP** | 1.081 | 0.685 | 5 | 9 |

N/A: No expression data available NP: Not published. Only significant zinc-regulated genes published in supplementary datasets

Black: Up-regulated ≥1.5 fold, p<0.05. Red: up-regulated <1.5 fold (equivalent to 0.585 log transformed) and/or p>0.05.

*Liver Organoid Media Formulation*

Organoids were cultured based on protocols developed by Broutier *et al.^1^.* Organoid expansion media was prepared fresh and stored at 4C for a maximum of 1 week prior to use. Expansion and differentiation media are listed below.

***Table S2. Expansion media recipe***

| **Expansion Media Component** | **Concentration** | **Supplier** | **Catalogue #** |
| --- | --- | --- | --- |
| **Advanced DMEM/f12** | 1x | Life Technologies | 12634010 |
| **Glutamax** | 1x | Life Technologies | 35050061 |
| **HEPES** | 10mM | Sigma | H3375 |
| **B27 Supplement** | 1x | Life Technologies | 17504001 |
| **N2 Supplement** | 1x | Life Technologies | 17502001 |
| **n-Acetylcysteine** | 1mM | Sigma | A7250-5G |
| **Gastrin** | 10nM | Sigma | SCP0152 |
| **hEGF** | 50ng/ml | Peprotech | AF-100-15 |
| **HGF** | 25ng/ml | Peprotech | AF-100-39H |
| **A83-01** | 5uM | R&D Systems | 2939 |
| **R-Spondin** | 500ng/ml | Peprotech | 120-38 |
| **Nicotinamide** | 10mM | Sigma | 72340-100G |
| **Forskolin** | 10uM | Peprotech | 6652995 |
| **FGF10** | 100ng/ml | Peprotech | AF-100-26 |

***Table S3. Differentiation media recipe***

| **Differentiation Media Component** | **Concentration** | **Supplier** | **Catalogue #** |
| --- | --- | --- | --- |
| **Advanced DMEM/f12** | 1x | Life Technologies | 12634010 |
| **Glutamax** | 1x | Life Technologies | 35050061 |
| **HEPES** | 10mM | Sigma | H3375 |
| **B27 Supplement** | 1x | Life Technologies | 17504001 |
| **N2 Supplement** | 1x | Life Technologies | 17502001 |
| **n-Acetylcysteine** | 1mM | Sigma | A7250-5G |
| **Gastrin** | 10nM | Sigma | SCP0152 |
| **hEGF** | 50ng/ml | Peprotech | AF-100-15 |
| **HGF** | 25ng/ml | Peprotech | AF-100-39H |
| **A83-01** | 0.5uM | R&D Systems | 2939 |
| **BMP7** | 25ng/ml | Peprotech | 120-03P |
| **Dexamethasone** | 3uM | Peprotech | 5000222 |
| **DAPT** | 10uM | Peprotech | 2088055 |
| **FGF19** | 100ng/ml | Peprotech | AF-100-32 |

***Table S4. Primer sequences and TaqMan probes***

| **Gene** | **Catalogue # or Primer Sequence** | **Channel** |
| --- | --- | --- |
| MT1A | Hs00831826_s1 | FAM |
| MT1E | Hs01582977_gH | FAM |
| MT1F | Hs00744661_sH | FAM |
| MT1G | Hs02578922_gH | FAM |
| MT1H | Hs00823168_g1 | FAM |
| MT1M | Hs00828387_g1 | FAM |
| MT1X | Hs00745167_sH | VIC |
| MT2A | Hs02379661_g1 | VIC |
| SLC30A1 | Hs00253602_m1 | VIC |
| HMOX1 | Hs00157965_m1 | VIC |
| DDIT3 | Hs00358796_g1 | VIC |
| HSPA6 | Hs04187232_g1 | VIC |
| 36B4 | F- 5’GTCCTCGTGGAAGGCCC  R- 5’AGGAGAGACAGGGAGCTCAG | FAM (SYBR) |
| UBC | F- 5’CAGCCGGGATTTGGGTCG  R- 5’CACGAAGATCTGCATTGTCAAGT | FAM (SYBR) |
| ORM1 | F- 5' GAGTACCAGACCCGACAGGAC  R- 5' CCTGAGGATCAGCAAGTGAGC | FAM (SYBR) |
| C4BPA | F- 5' TGCCTGCTGTTCTTGGCAAT  R- 5'GGGATCTGACGTAGCCAGGG | FAM (SYBR) |

***Table S5. MTF-1 enrichment in zinc-stimulated gene sets.***

| **Gene Sets** | **Z Score** | **P Value** | **Bonferroni p-value** | **Sample Average** | **Background Average** | **Genes** |
| --- | --- | --- | --- | --- | --- | --- |
| Zinc Signature | 8.95524 | 1.68E-19 | 1.25E-16 | 0.848464 | 0.72891 | MT1F, SLC30A1, MT1H, MT2A, MT1X, MT1G, MT1E, MT1A, SOCS2, MT1M |
| ZSG ≥5 Datasets | 7.02978 | 1.00E-12 | 7.48E-10 | 0.795306 | 0.72891 | MT1F, SLC30A1, MT1H, MT2A, MT1X, MT1G, MT1E, MT1A, SOCS2, MT1M, HSPA6, HMOX1, BAG3, DNAJB1, DDIT3, TRIB3, CDKN1A, ASNS |
| ZSG ≥4 Datasets | 7.86411 | 1.63E-15 | 3.59E-12 | 0.770713 | 0.72891 | MT1F, SLC30A1, MT1H, MT2A, MT1X, MT1G, MT1E, MT1A, SOCS2, MT1M, HSPA6, HMOX1, BAG3, DNAJB1, DDIT3, TRIB3, CDKN1A, ASNS, EGR1, RGS16, ZFAND2A, HSPH1, RASD1, HBEGF, PPP1R15A, DUSP5, EMP1, JUN, FKBP4, RIMS3, FOS, KLF4, WBP5, RGS2, KLF6, CSRP1, ASB13, HSPA1B, MTE, SLC3A2 |
| ZSG ≥3 Datasets | 6.21016 | 2.31E-10 | 1.72E-07 | 0.751789 | 0.72891 | MT1F, SLC30A1, MT1H, MT2A, MT1X, MT1G, MT1E, MT1A, SOCS2, MT1M, HSPA6, HMOX1, BAG3, DNAJB1, DDIT3, TRIB3, CDKN1A, ASNS, EGR1, RGS16, ZFAND2A, HSPH1, RASD1, HBEGF, PPP1R15A, DUSP5, EMP1, JUN, FKBP4, RIMS3, FOS, KLF4, WBP5, RGS2, KLF6, CSRP1, ASB13, HSPA1B, MTE, SLC3A2, FOSB, ATF3, GRHL1, ETS1, DEDD2, MAFF, CHAC1, ARL14, DHDH, TES, SERPINH1, SESN2, RN7SK, ZFP36, ABTB2, P4HA2, NAB2, MAMLD1, TMEM158, SDC4, ARID5B, DNAJB6, UBC, EFNB2, SPIRE1, IRAK2, GRB10, TRIB1, GABARAPL1, TNFRSF9, TSHZ1, FHL2, RND3, PLAUR, CHSY1, DUSP1, PMAIP1, ULBP2, TNFRSF12A, NDRG1, ARL5B, HSPD1, HES4, HSPA4, RIOK3, SLC2A3, EHD1, ABLIM1, SERTAD1, SEMA4B, PHLDA1, SPSB1, TXNRD1, P4HA1, CSRP2, STC2, SOCS3, SPRED2, TP53INP2, VEGFA, SPAG9, DDIT4, GADD45B, GDF15, PLAGL2, RAPGEFL1, OTUD1, TSPYL2, CNBP, SLC30A2, ENDOGL1, SLC7A11, TUBB3, HSPA1A, LOC441019, MT1B, CRISPLD2, ADM, IER5, MT1HL1, MTHFD2, BTG2, CNKSR3, SPP1 |

***Table S6. Liver disease datasets***

| **Aetiology** | **DataSet** | **Host** | **Samples** | **Figure** |
| --- | --- | --- | --- | --- |
| **HBV** | GSE83148 | NCBI GEO | 6 Control vs 122 HBV+ | Fig. 4A |
|  |  |  |  |  |
| **HCV** | GSE15331 | NCBI GEO | 6 HCV- vs 24 HCV+ | Fig. 4A |
|  | GSE70779 | NCBI GEO | 9 HCV cured vs 9 HCV+ | Fig. 4A |
|  |  |  |  |  |
| **NAFLD** | GSE135251 | NCBI GEO | 10 Control vs 51 NAFLD | Fig. 4A |
|  | GSE48452 | NCBI GEO | 10 Control vs 14 Steatosis | Fig. 4A |
|  | GSE66676 | NCBI GEO | 34 Control vs 26 NAFLD | Fig. 4A |
|  | PRJNA512027 | NCBI BioProject | 36 Control vs 50 Grade 2/3 Steatosis | Fig. 4A |
|  |  |  |  |  |
| **NASH** | E_MEXP_3291 | EMBL_EBI | 19 Control vs 16 NASH | Fig. 4A |
|  | GSE135251 | NCBI GEO | 10 Control vs 34 NASH | Fig. 4A |
|  | GSE167523 | NCBI GEO | 51 NAFLD vs 47 NASH | Fig. 4A |
|  | GSE48452 | NCBI GEO | 14 Control vs 18 NASH | Fig. 4A |
|  | GSE66676 | NCBI GEO | 34 Control vs 7 NASH | Fig. 4A |
|  |  |  |  |  |
| **HCC** | GSE14520 | NCBI GEO | 220 Non-Tumour vs 225 Tumour | Fig. 4A |
|  | GSE25097 | NCBI GEO | 243 Non-Tumour vs 268 Tumour | Fig. 4A |
|  | GSE84402 | NCBI GEO | 14 Adjacent Healthy vs 14 Tumour | Fig. 4A |
|  | GSE14323 | NCBI GEO | 19 Control vs 38 Tumour | Fig. 4A |
|  |  |  |  |  |
| **Cirrhosis** | GSE14323 | NCBI GEO | 19 Control vs 41 Cirrhosis | Fig. 4A |
|  | GSE135251 | NCBI GEO | 10 Control vs 14 Cirrhosis | Fig. 4A |
|  | GSE49541 | NCBI GEO | 40 mild fibrosis (F0-1) vs 32 Advanced (F3-4) | Fig. 4A |
|  | PRJNA512027 | NCBI BioProject | 36 Control vs 18 Cirrhosis | Fig. 4A |
|  | GSE25097 | NCBI GEO | 6 Control vs 40 Cirrhotic | Fig. 4A |
| **ALD/AH** | GSE28619 | NCBI GEO | 7 Control vs 15 Severe AH | Fig. 4B |
|  | GSE143318 | NCBI GEO | 5 Control vs 5 Severe AH | Fig. 4B |
|  | GSE155907 | NCBI GEO | 4 Control vs 6 Severe AH | Fig. 4B |
|  | GSE142530 | NCBI GEO | 12 Control vs 10 Severe AH | Fig. 4B |
|  | GSE94397 | NCBI GEO | 71 AH | Fig. 5 |
|  | GSE94399 | NCBI GEO | 38 AH | Fig. 5 |
|  | GSE103580 | NCBI GEO | 110AH | Fig. 5 |
| **Other** | [phs003112.v1.p1](https://www.ncbi.nlm.nih.gov/projects/gap/cgi-bin/study.cgi?study_id=phs003112.v1.p1) | dbGaP | 10 Control, 10 MAFLD, 19 HCV, 41 AH | Fig. 4C/D/E |

**Supplementary Results**

**
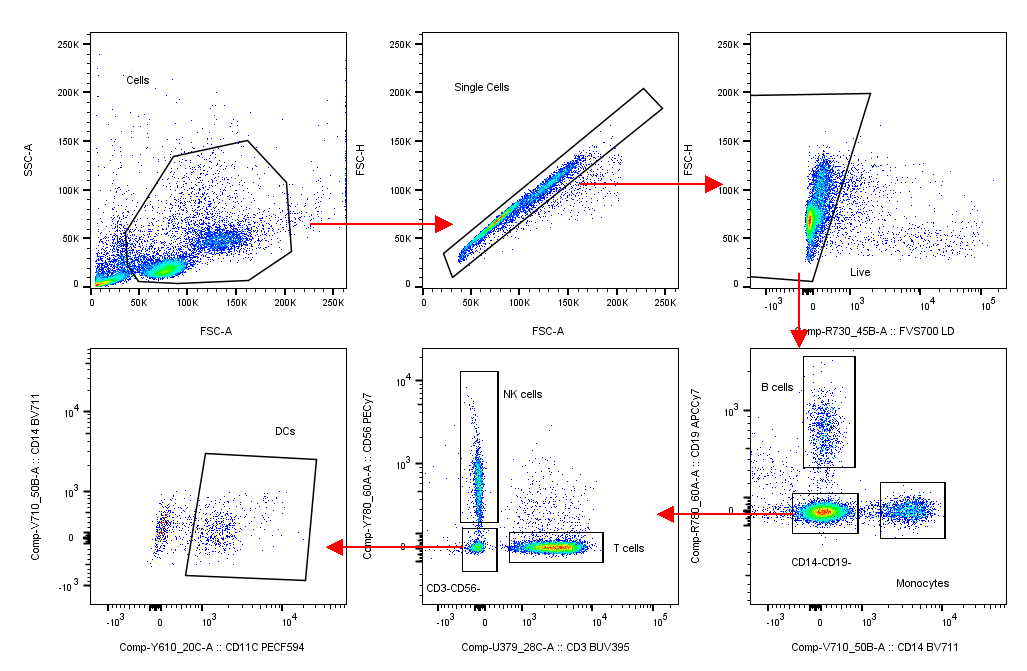
**

**Figure S3. Peripheral blood mononuclear cell gating.** Live single cells were gated into CD14+CD19- monocytes and CD14-CD19+ B cells, after which double negative cells were gated into CD56-CD3+ T cells and CD56+CD3- T cells. The double negative population was then gated into CD11c+ dendritic cells.


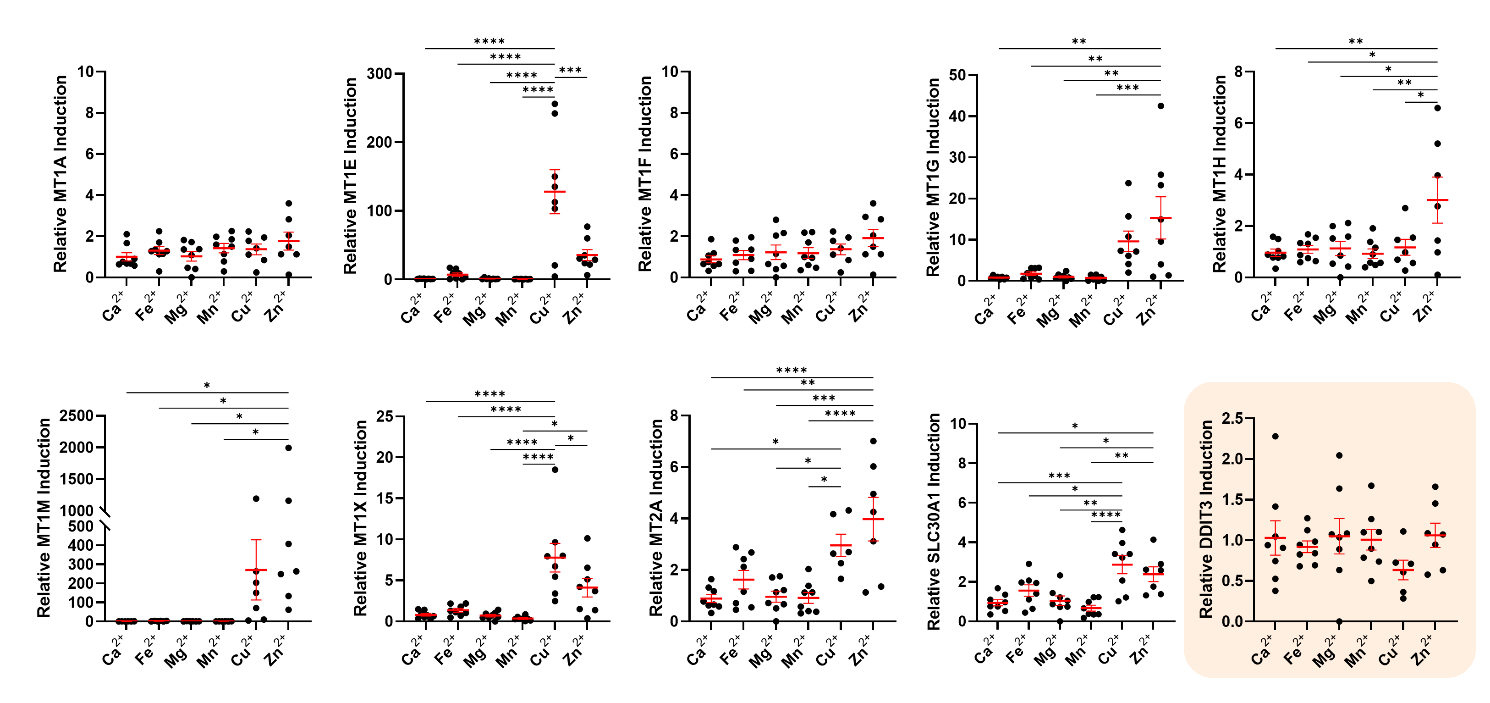


**Figure S4. Induction of zinc-responsive genes following treatment with divalent cations.** Peripheral blood mononuclear cells were treated with 50 µM metal salts containing divalent cations calcium (Ca^2+^), copper (Cu^2+^) manganese (Mn^2+^), magnesium (Mg^2+^) and iron (Fe^2+^) in addition to zinc (Zn^2+^) for 24 h and zinc-stimulated genes were measured by qPCR. Repeated measures one way ANOVA, * p<0.05, ** p<0.01, ***p<0.001, **** p<0.0001.

**References**

1. Broutier, L. *et al.* Culture and establishment of self-renewing human and mouse adult liver and pancreas 3D organoids and their genetic manipulation. *Nat Protoc* **11**, 1724-1743 (2016).
